# Fast Quantum Gates with Electric Field Pulses and Optical Tweezers in Trapped Ions

**DOI:** 10.3390/e27060595

**Published:** 2025-05-31

**Authors:** Clara Robalo Pereira, Liam J. Bond, Matteo Mazzanti, Rene Gerritsma, Arghavan Safavi-Naini

**Affiliations:** 1 Institute for Theoretical Physics, Institute of Physics, University of Amsterdam, Science Park 904, 1098 XH Amsterdam, The Netherlands; m.c.robalopereira@uva.nl (C.R.P.); l.j.bond@uva.nl (L.J.B.); a.safavinaini@uva.nl (A.S.-N.); 2 Van der Waals-Zeeman Institute, Institute of Physics, University of Amsterdam, 1098 XH Amsterdam, The Netherlands; 3 QuSoft, Science Park 123, 1098 XG Amsterdam, The Netherlands; 4 Institute for Quantum Electronics, ETH Zürich, Otto-Stern-Weg 1, 8093 Zürich, Switzerland; mmazzanti@phys.ethz.ch; 5 Quantum Center, ETH Zürich, 8093 Zürich, Switzerland

**Keywords:** optical tweezers, quantum computation, trapped ions, fast gate

## Abstract

We propose a two-qubit phase gate based on trapped ions that uses fast electric field pulses and spin-dependent local traps generated by optical tweezers. The phases are engineered by spin-dependent coherent evolution, interspersed with momentum kicks. We derive a set of commensurability conditions and expressions for the spin-dependent accumulated phase that, when satisfied, realize the target two-qubit phase gate within tens of microseconds. We study the scalability of our proposal in larger-ion crystals and demonstrate the existence of solutions with up to four ions. Gates in larger crystals should also be possible but will require more commensurability conditions to be fulfilled.

## 1. Introduction

Trapped ions are one of the leading platforms for quantum computing. Qubits encoded in individual ions can be very precisely manipulated and addressed and feature long coherence times [1,2,3]. However, especially as the algorithm complexity increases, there is a demand for calculations with a higher number of operations. It is then of importance to develop schemes for high-speed and high-fidelity gates, which can outpace decoherence, whilst maintaining robustness. The goal is to obtain short operational times which protect the system against heating and other sources of decoherence. This has led to the development of faster gate schemes [4,5,6,7], which rely on non-adiabatic excitations of motional modes. In this way, gate operations can achieve gate speeds faster than one oscillation period.

Previous fast gates schemes have considered lasers for generating the state-dependent force needed to couple motional states and spin-states [4,8]. Such proposals have been realized in experiments [9,10], as well as generalized to large ion arrays [5,11,12] and microtraps [13]. However, laser-based kicks require exceptional control over the timing and phase coherence of laser pulses with high power. This is challenging to realize in experiments and scales poorly with the ion number. An alternative proposition is the use of Rydberg ions [14], in which the state dependence derives from the difference in the trapping potential experienced due to the large polarizability of the Rydberg states. Another option is to use trapped ions interacting with an optical lattice [15].

**Figure 1:**
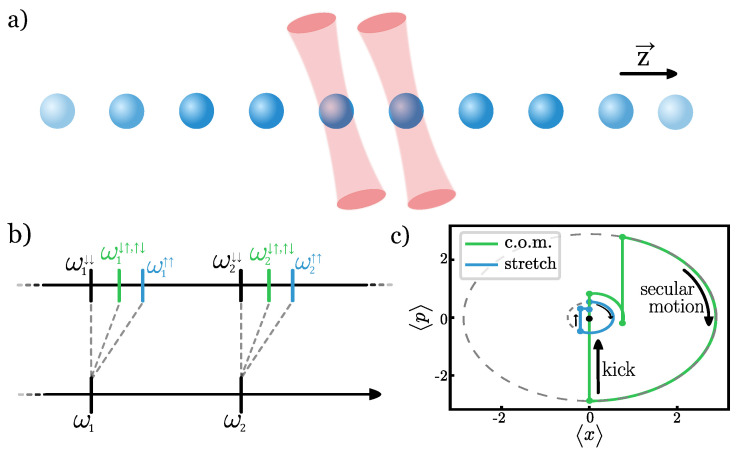
The setup required for the proposed phase gate. (**a**) An ion chain with tweezers pinning the two target ions. (**b**) The tweezers modify the phonon spectrum spin-dependently. (**c**) The illustrated spin-dependent phase space trajectory for an arbitrary spin-state. The coherent evolution depends on the mode frequencies, and the commensurability conditions ensure that the phase space loops are closed. The accumulated phase equals the area of each phase loop.

In this work, we propose a different mechanism for generating the qubit state-dependent force: optical tweezers that generate a state-dependent potential localized around each ion. This extends our recent work in [16] to the non-perturbative regime, where the tweezer potential modifies not only the mode frequencies but also the normal mode structure [17,18,19,20]. This allows us to realize a faster gate implementation that is more robust to decoherence. An illustration of the gate mechanism applied to a two-qubit crystal is depicted in Figure 1.

Our scheme is an extension of the scheme of Ref. [4]. It uses momentum kicks applied at specific times generated by an electric field, interspersed with tweezer-modified coherent evolution for predetermined durations, such that the phase accumulated by each two-qubit state is the appropriate phase required by the target logic gate [4]. Furthermore, we require that the motional modes follow closed trajectories in phase space such that the initial state is recuperated, effectively decoupling the electronic and motional states by the end of the gate [21]. The set of commensurability conditions which must be satisfied in order to achieve this scales as $2^{2}N_{i}$, where $N_{i}$ is the number of ions in the crystal. Clearly, with increasing ion numbers, it becomes increasingly hard to exactly satisfy these conditions, but an optimal solution can be found numerically in large-ion crystals.

## 2. System Description: Two Ions

Consider $N_{i}=2$ ions in a one-dimensional harmonic trap with the trapping frequency ω. The motion of the ions is described by

(1) $$\begin{array}{c} H_{0}=\hbar \sum_{m}\omega_{m}\left(a_{m}^{†}a_{m}+1/2\right), \end{array}$$

where $a_{m}$ ($a_{m}^{†}$) are the bosonic annihilation (and creation) operators for the center of mass (com) and stretch modes with the corresponding mode frequencies $\omega_{c}=\omega$ and $\omega_{r}=\sqrt{3}\omega$, respectively.

We consider state-dependent optical tweezers focused at the equilibrium positions of the ions, each with a local trapping frequency $\omega_{\mathrm{tw}}∼\omega_{m}$

(2) $$\begin{array}{c} {\hat{H}}_{\mathrm{tw}}=\frac{1}{2}m\omega_{\mathrm{tw}}^{2}\sum_{i=1,2}{\hat{z}}_{i}^{2}{\left|↑\rangle \langle ↑\right|}_{i}, \end{array}$$

where we have used the harmonic approximation for the Gaussian tweezer potential. Here, *m* is the mass of the ion, and ${\hat{z}}_{i}$ is the position of the *i*th ion with respect to its equilibrium position. Equation (2) is a state-dependent potential that mixes the native com and stretch modes, resulting in new state-dependent modes $b_{m}^{\cdot \cdot }$ with the corresponding frequencies $\omega_{m}^{\cdot \cdot }$ and creation (annihilation) operators ${a_{m}^{\cdot \cdot }}^{†}$ ($a_{m}^{\cdot \cdot }$). We use $\cdot_{1},\cdot_{2}\in \left\{↑,↓\right\}$ to represent the internal state of the first and second ions, respectively. We use this notation throughout to denote that a term is state-dependent.

Finally, we require a force F generated by an instantaneous, spatially uniform electric field E that gives a momentum kick to the ions:

(3) $$\begin{array}{c} {\hat{H}}_{\mathrm{kick}}\left(F\right)=F\cdot \hat{z}, \end{array}$$

where $\hat{z}=\left\{{\hat{z}}_{1},{\hat{z}}_{2}\right\}$, such that the elements of F describe the force on each ion.

The gate sequence we consider consists of two evolution steps applied *N* times: (i) evolution under ${\hat{H}}_{\mathrm{free}}={\hat{H}}_{0}+{\hat{H}}_{\mathrm{tw}}$ for a duration $\tau_{k}$, followed by (ii) a kick generated by ${\hat{H}}_{\mathrm{kick}}$ with the force $F_{k}$ applied at time $t_{k}$ for a time δt→0, where *k* references the kick sequence number. The evolution of the total sequence is therefore given by $U_{\mathrm{total}}=\prod_{k=1}^{N}U_{\mathrm{kick}}^{\left(k\right)}U_{\mathrm{free}}^{\left(k\right)}$.

To characterize the protocol, we consider the action of the unitary on coherent states in the presence of the tweezers $|\cdot \;\cdot ,\alpha_{1}^{\cdot \cdot },\alpha_{2}^{\cdot \cdot }\rangle$, where $|\alpha_{1}^{\cdot \cdot }\rangle$ ($|\alpha_{2}^{\cdot \cdot }\rangle$) is the spin-dependent coherent state of the com (stretch) mode. Such states can be prepared by cooling the motional modes in the absence of tweezers to prepare the ground state |0〉, before adiabatically ramping the tweezer’s strength for approximately one trap period to prepare $|0^{\cdot \cdot }\rangle$. We describe this in more detail in Appendix A.

The action of the free evolution operator is

(4) $$\begin{array}{cc} U_{\mathrm{free}}^{\left(k\right)}|\cdot \;\cdot ,\alpha_{1}^{\cdot \cdot },\alpha_{2}^{\cdot \cdot }\rangle \; & =e^{-i\sum_{m}\omega_{m}^{\cdot \cdot }\left({a_{m}^{\cdot \cdot }}^{†}a_{m}^{\cdot \cdot }+1/2\right)\tau_{k}}|\cdot \;\cdot ,\alpha_{1}^{\cdot \cdot },\alpha_{2}^{\cdot \cdot }\rangle \rangle \\ \; & =e^{-\left(i/2\right)\left(\omega_{1}^{\cdot \cdot }+\omega_{2}^{\cdot \cdot }\right)\tau_{k}}|\cdot \;\cdot ,e^{-i\omega_{1}^{\cdot \cdot }\tau_{k}}\alpha_{1}^{\cdot \cdot },e^{-i\omega_{2}^{\cdot \cdot }\tau_{k}}\alpha_{2}^{\cdot \cdot }\rangle . \end{array}$$

The electric field kick can be expanded by substituting in the position operator $\hat{z}=\sum_{m}l_{m}^{\cdot \cdot }\left({a_{m}^{\cdot \cdot }}^{†}+a_{m}^{\cdot \cdot }\right)b_{m}^{\cdot \cdot }$, yielding

(5) $$U_{\mathrm{kick}}^{\left(k\right)}=\exp\left(-i\sum_{m}l_{m}^{\cdot \cdot }\left(b_{m}^{\cdot \cdot }\cdot F_{k}\right)\left({a_{m}^{\cdot \cdot }}^{†}+a_{m}^{\cdot \cdot }\right)\delta t/\hbar \right),$$

where $l_{m}^{\cdot \cdot }=\sqrt{\hbar /2M\omega_{m}^{\cdot \cdot }}$ is the (spin-dependent) harmonic oscillator length scale. The kick operator is thus simply a displacement operator such that

(6) Ukick(k)|··,α1··,α2··〉=exp(−i∑mpm,k··Re{αm})|··,α1−ip1,k··,α2−ip2,k··〉,

where we have introduced $p_{m,k}^{\cdot \cdot }=F_{k}\delta tl_{m}^{\cdot \cdot }\left({\tilde{b}}_{m}^{\cdot \cdot }\cdot 1_{2}\right)/\hbar$, which is in the order of unity, with $1_{2}$ the two-element vector (1,1).

Finally, the total unitary produces $U_{\mathrm{total}}|\cdot \;\cdot ,\alpha_{1}^{\cdot \cdot },\alpha_{2}^{\cdot \cdot }\rangle =e^{i\xi }|\cdot \;\cdot ,{\tilde{\alpha }}_{1}^{\cdot \cdot },{\tilde{\alpha }}_{2}^{\cdot \cdot }\rangle$, with

(7) ξ=∑mΘm··−θN··2−∑n=2NReαm··∑k=1Npm,k··e−iθn··,

and

(8) $$\begin{array}{c} {\tilde{\alpha }}_{m}^{\cdot \cdot }=\alpha_{m}^{\cdot \cdot }e^{-i\theta_{N}^{\cdot \cdot }}-i\sum_{k=1}^{N}p_{m,k}^{\cdot \cdot }e^{-i(\theta_{k}^{\cdot \cdot }-\theta_{N}^{\cdot \cdot })}, \end{array}$$

where we have defined the phases $\Theta_{m}^{\cdot \cdot }=-\sum_{n=2}^{N}\sum_{k=1}^{n-1}p_{m,n}^{\cdot \cdot }p_{m,k}^{\cdot \cdot }\sin\left(\omega_{m}^{\cdot \cdot }\left(t_{k}-t_{n}\right)\right)$ and $\theta_{k}^{\cdot \cdot }=\omega_{m}^{\cdot \cdot }\sum_{n=1}^{k}\tau_{n}$.

To perform a closed loop in phase space, we require that ${\tilde{\alpha }}_{m}^{\cdot \cdot }=\alpha_{m}^{\cdot \cdot }$ for all two-qubit states. From Equation (8), we therefore require that

(9) $$\begin{array}{c} C_{m}^{\cdot \cdot }\equiv \sum_{k=1}^{N}p_{m,k}^{\cdot \cdot }e^{-i\omega_{m}^{\cdot \cdot }t_{k}}=0, \end{array}$$

where we have made the substitution $\sum_{n=1}^{k}\tau_{n}=t_{k}$, as well as either

(10a) $$\begin{array}{cc} \alpha_{m}^{\cdot \cdot } & =0,\;\mathrm{or} \end{array}$$

(10b) $$\begin{array}{cc} e^{i\theta_{N}^{\cdot \cdot }} & =1. \end{array}$$

Satisfying Equations (9) and (10a)—we will call this protocol 1—requires preparing the tweezer ground state and means that the accumulated phase depends on the zero-point energy term, so that Equations (7) and (8) can be simplified into

(11) $$\begin{array}{c} \xi \equiv \sum_{m}\left(\Theta_{m}^{\cdot \cdot }-\frac{\theta_{N}^{\cdot \cdot }}{2}\right), \end{array}$$

In contrast, satisfying Equations (9) and (10b), which we will call protocol 2, makes the gate independent of the initial motional state amplitude, ${\tilde{\alpha }}_{m}^{\cdot \cdot }=\alpha_{m}^{\cdot \cdot }$, and removes the zero-point energy term from the accumulated phase, so that the phase now becomes

(12) $$\begin{array}{c} \xi \equiv \sum_{m}\Theta_{m}^{\cdot \cdot }. \end{array}$$

Protocol 1 is easier in computational terms; however, it requires the system to be cooled down to the ground state. Protocol 2, conversely, is more powerful as it provides temperature-insensitive solutions, but it is much harder to satisfy, as it requires that the zero-point energy term, Equation (10b), is in phase for all four spin combinations. In this article, we consider the implementation of protocol 1. The commensurability conditions, Equation (9), mean we have, for each of three (four different spin combinations exist, but the eigenvalues of |↑↓〉 and |↓↑〉 are identical for a two-ion crystal) possible spin combinations |··〉, two normal modes *m* and for each of these *N* sets of kicks, totaling six equations. If we are to add the requirement for temperature insensitivity, Equation (10b), the fact that this term is spin-dependent means that we will necessarily be adding another six equations to our total. This, in turn, will scale with the number of ions in the gate, as adding one ion means adding one motional mode to the system. In order to perform the logic gate, we must not only satisfy these conditions but must also guarantee that the overall phase accumulated over the system’s evolution realizes the desired phase gate:

(13) $$\begin{array}{c} \xi =\sum_{m}-\theta_{N}^{\cdot \cdot }/2+\Theta_{m}^{\cdot \cdot }+\Xi_{m}^{\cdot \cdot }=\frac{\pi }{4}\left(\begin{array}{cccc} 1 & \; & & \\ \; & -1 & \; & \\ \; & \; & -1 & \\ \; & \; & & 1 \end{array}\right). \end{array}$$

Full expressions for $\Xi^{\cdot \cdot }$ and $\Theta^{\cdot \cdot }$ for each of the four different possible qubit states can be found in Appendix E.

## 3. Results

In order to implement the phase gate, we search for solutions of the type $\left(z_{k}\equiv F_{k}\delta t,\tau_{k}\right)$, with *k* denoting each sequence step. Besides satisfying the commensurability conditions and the overall phase constraint, we choose the total number of steps, *N*, and also constrain the momentum amplitude of the kicks $z_{k}$ in each step or the total gate time $T_{\mathrm{gate}}$. To facilitate this search, spin-echo sequences could potentially be performed at any point in the sequence to eliminate dephasing between states and facilitate the search for an adequate gate sequence.

An example sequence can be observed in Figure 1c, which represents a two-ion phase gate for on a two-ion crystal, with the tweezers pinning the two ions in the array. The kicks represent pure displacements in momentum space, and the free evolution periods are secular motion in 〈x,p〉 space with a constant phase. The initial phase space orbit for each of the normal modes is restored by the end of the pulse sequence, demonstrating that the commensurability conditions are satisfied.

In order to quantify the gate performance, we investigate the average gate fidelity [22]:

(14) $$\begin{array}{c} \bar{F}\left(E,U\right)=\frac{\sum_{j}\mathrm{Tr}\left(UU_{j}^{†}U^{†}E\left(U_{j}\right)\right)+d^{2}}{d^{2}\left(d+1\right)}, \end{array}$$

where U is the target gate operation, E is our generated unitary, and $E\left(U_{j}\right)=tr_{\mathrm{FS}}[E(|n\rangle \langle n|⊗\;U_{j})E]$ is its projection on the motional modes $|\alpha_{i}\rangle$ and on the SU(2) generalized Pauli matrices, $U_{j}$, of dimension d, with d=4 for two-ion gates.

**Figure 2:**
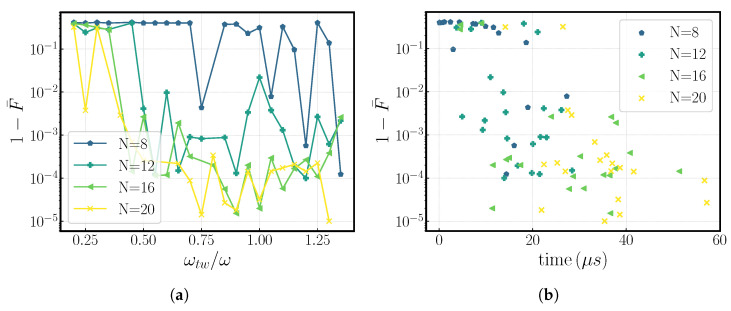
Gate infidelity, $1-\bar{F}$, as a function of (**a**) tweezer frequency, $\omega_{\mathrm{tw}}$, and (**b**) overall gate time for a two-ion crystal. These results were obtained by performing a search over different possible kick number values in a range pf 8≤N≤20. We observe that the gate is harder to perform at lower tweezer frequencies, which is the reason why below $\omega_{\mathrm{tw}}/\omega$ = 0.5, solutions were found only in the region $1-\bar{F}>10^{-4}$. In addition, small kick numbers, despite resulting in smaller gate times, usually result in higher infidelities as the number of free parameters is reduced, whereas longer gate times yield more solutions with better infidelities, at the cost of the overall gate time. The infidelity is presented on a logarithmic scale.

After sweeping over different values for the kick number, *N*, and tweezer frequency, $\omega_{\mathrm{tw}}$, we obtain the results shown in Figure 2 for a gate performed in a two-ion crystal. The infidelities obtained are as good as $1-\bar{F}∼\left[10^{-5}\right]$, so long as we have an adequate number of kicks (generally N>12), and the employed tweezer frequency is $\omega_{\mathrm{tw}}/\omega \geq 0.5$; see Figure 2a.

Larger numbers of kicks, as in Figure 2a,b, $N_{i}$, generally translate into lower achievable infidelities regardless of the tweezer frequency but at the expense of the overall gate time $T_{\mathrm{gate}}$. We attribute this trend to the added degrees of freedom which a higher number of kicks introduces into the problem, thus increasing the solution space and the probability of finding converging solutions. In general, we conclude that having higher tweezer frequencies means lower infidelities can be achieved, requiring smaller kick numbers and thus shorter gate times; see Figure 2b.

### Gates in a Four-Ion Crystal

**Figure 3:**
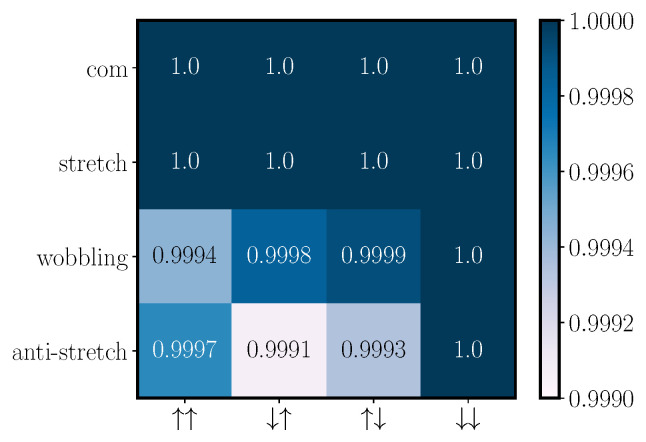
The overlap between the initial and final motional states for a gate performed in a four-ion crystal, with tweezers pinning the two left-most ions, using a tweezer frequency $\omega_{\mathrm{tw}}=0.8\omega$ and N=16 kicks. The gate takes 37μs and has an average gate fidelity of $\bar{F}=0.996$. For each mode and spin combination pair, we plot the coherent state overlap between the initial (before gate) and final (after gate) state configurations, $\langle \alpha_{i}|\alpha_{f}\rangle$.

**Figure 4:**
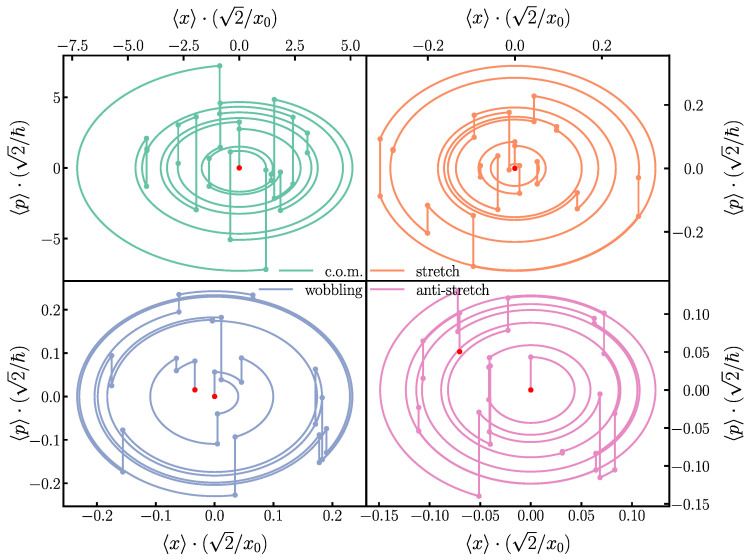
The phase space trajectories of the spin-state |↓↑〉 for the four motional modes in a 4-ion crystal, performing the gate on the 2 left-most ions. These trajectories correspond to the same gate used in Figure 3, with a tweezer frequency $\omega_{\mathrm{tw}}=0.8\omega_{z}$ and N=16 kicks. It can be observed in the **lower-right** and **-left** panels how the wobbling and anti-stretch modes do not exactly close at the end of the trajectory.

We investigate the scalability of this scheme by increasing the number of ions in the crystal to four. Even though the gate is performed solely between two ions, the electric field kicks must be determined so that the new additional motional modes also recover their initial states. The additional constraints hinder the convergence of our numerical methods such that it is harder to find solutions. The solutions found do not perform as well as in the two-ion case; we observe an overall reduction in fidelity: the produced unitary does not match the desired target phase gate as well as it does in the two-ion case, and for some motional mode/spin-state combinations, the overlap between the initial and final states is not exact; see Figure 3. Another way to visualize the overlap between the initial and final states is to look at the phase space trajectories of the different modes throughout the gate and compare the final phase space position with the initial one; see Figure 4.

Even though we observe a reduction in performance on scaling up the ion crystal size, it is important to note that a beneficial aspect in the scalability of this gate scheme is that the relative extent of the excursions in the phase space is reduced for higher-frequency modes; see Figure 4. This happens because the electric field only couples to the center-of-mass character of the mode, and this favors the low-frequency modes in ions. When adding ions to a crystal, the number of modes increases through ‘adding’ higher-frequency modes, while the modes that were already there remain unchanged. So, while we add error by adding ions, the amount of error that is added per ion reduces. It is therefore conceivable that not all modes have to be taken into account for large-ion crystals, at the expense of a small but unavoidable error in the gate.

To conclude, we can successfully find four-ion crystal solutions with average phase gate infidelities as good as $1-\bar{F}∼10^{-4}$, though more commonly with $1-\bar{F}∼10^{-2}$; see Table 1.

Together with the results in Figure 2, these results lead us to believe that the choice of tweezer frequency does not condition our scheme in a monotonous way (meaning there is not a clear trend in how it affects the gate performance), but rather it conditions our results in two ways: one, it defines how strongly our states are coupled to motion and how much our eigenfrequencies are modified. So, with a higher tweezer frequency, it is easier to accumulate the desired phases with fewer kicks, whereas lower tweezer frequencies require more kicks; see Figure 2a). Secondly, while studying the four-ion case, we realize that it is easier to perform multiple-ion gates (>two ions) using lower tweezer frequencies, a fact justified due to the tweezer inducing lower state mixing, which reduces the amount of dephasing gathered at each step by the different motional states.

## 4. Experimental Considerations

The electric field’s amplitude $E_{0}$ and pulse duration δt represent experimental bottlenecks in terms of gate feasibility. To justify the assumption of instantaneous kicks, the electric field pulses must be much faster than the motion of the ion in the trap. Since typical trap frequencies lie in the MHz range, we must set δt≪ μs. We can estimate the typical electric fields required by setting $l_{m}eE_{0}\delta t/\hbar =1$. For electric field pulse durations in the 10–100 ns range, we calculate $E_{0}∼$ 1–10 V/m, with a slight dependence on the ion species. Our results yield electric field requirements in the order of $∼\left\{10^{-6},10^{-7}\right\}\left[\mathrm{Vs}/m\right]$, which are thus experimentally feasible.

**Figure 5:**
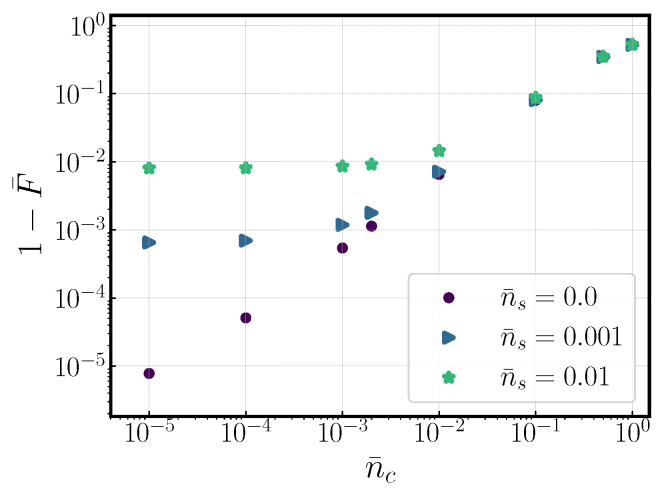
Infidelity as a function of com mode number occupation, ${\bar{n}}_{c}$, for gates originally with infidelities ∼$10^{-6}$ and three different stretch mode occupations, ${\bar{n}}_{s}$. The gate solution used to generate this graph employed seven kicks and had a gate time of ∼23μs.

In this article, we have stayed within the confines of protocol 1, which requires ground state cooling and for Equation (10a) to be satisfied. In this way, it is relevant to investigate how the gate performs if this cooling is not perfect and there is some mode occupation beyond the ground state. These results are portrayed in Figure 5, where an originally $1-\bar{F}=10^{-6}$ solution is exposed to increasing com. mode occupation, ${\bar{n}}_{c}$, for three different values of occupation of the stretch mode, ${\bar{n}}_{s}$. We observe how the infidelity decays to $10^{-2}$ as the com number occupation rises to the same value, following the same exponential trend. The number occupation of the stretch mode equally limits the minimum achievable infidelity.

Finally, it can be experimentally challenging to generate a large difference in polarizability between the two qubit states. Here, one approach is to employ optical qubits in which one of the qubit states is encoded in a metastable electronic state. For instance, we can consider the qubit states $\left|0\rangle =\right|S_{1/2},m_{j}=1/2\rangle$ and $\left|1\rangle =\right|D_{5/2},m_{j}=3/2\rangle$ in Ca+40 [23]. We can use a tweezer at 532 nm with circular $\sigma^{+}$ polarization. Setting the waist to 1μm and using a modest power of P= 1 mW generates a tweezer trap frequency of $\omega_{\mathrm{tw}}=2\pi \times$ 49.1 (2.7) kHz for the state |0〉 (|1〉). We calculate the photon scattering to be γ≲ $1s^{-1}$. Note that the photon scattering may be reduced further by employing hollow tweezers, allowing for larger $\omega_{\mathrm{tw}}$ values. For these calculations, we took into account the main dipole-allowed transitions and used the energies and transition dipole moments reported in [24].

## 5. Conclusions and Outlook

We proposed a two-qubit gate scheme on an array of trapped ions which relied on optical tweezers to generate the state dependence. Electric field pulses generate momentum kicks which produce the desired phases and accelerate the phase space trajectories. We calculate that the gate can be performed within accessible experimental conditions, with the caveat being that ground state cooling is required in the studied regime. We obtain gate times that are up to two orders of magnitude faster than those in the case studied in [16] but still fall short of the oscillation period of the ions in their trap [4]. This is due to the fact that this scheme requires dealing with the excitation of all modes, whose excursions in phase space must be undone, an effort which is leveraged against the speed of the fast pulses.

Although the proposed scheme may be easier to implement than either schemes based on pulsed lasers or Rydberg ions, it is limited by the challenge of supplying large state-dependent potentials to the ions without significant photon scattering. We note that the use of hollow tweezers would offer benefits in this regard [16,25,26]. We study how well the scheme performs as we scale up to a four-ion crystal, finding infidelities generally two orders of magnitude below the two-ion crystal solutions. Finally, a different search strategy or problem formulation could yield temperature-independent solutions, making the scheme more accessible under realistic experimental conditions.

## Figures and Tables

**Table 1:** Average phase gate infidelity and gate time for several four-ion solutions. We present solutions with different kick numbers for a tweezer frequency $\omega_{\mathrm{tw}}=0.4\omega$ and one sixteen-kick solution for a tweezer frequency $\omega_{\mathrm{tw}}=0.8\omega$.

$\omega_{\mathrm{tw}}/\omega$	N	$1-\bar{F}$	$T_{\mathrm{gate}}$ (μs)
0.4	16	0.0373	22.5717
0.4	20	0.0100	17.2863
0.4	28	0.0402	41.5518
0.4	30	0.0002	16.3239
0.8	16	0.0039	37.0045

**Figure A1:**
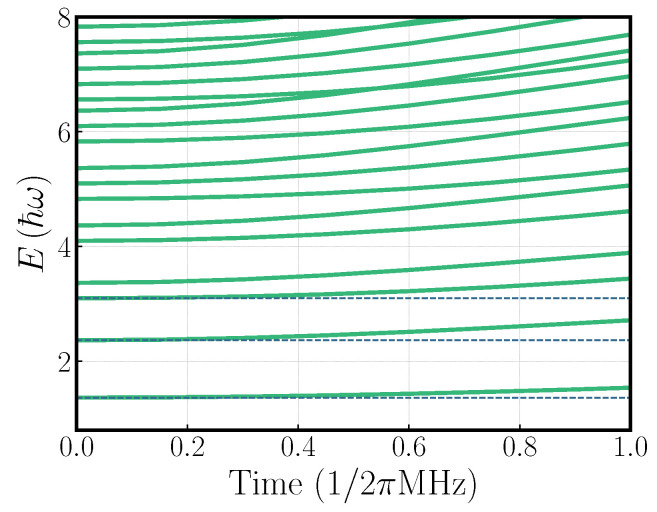
The dependence of the energy spectrum of the system on tweezer pinning frequency $\omega_{\mathrm{tw}}/\omega$. Level crossings are visible at higher energies due to the change in com mode frequency scaling differing to that in the stretch mode. The three dashed lines correspond to the first three energy eigenvalues; see Appendix F.

**Figure A2:**
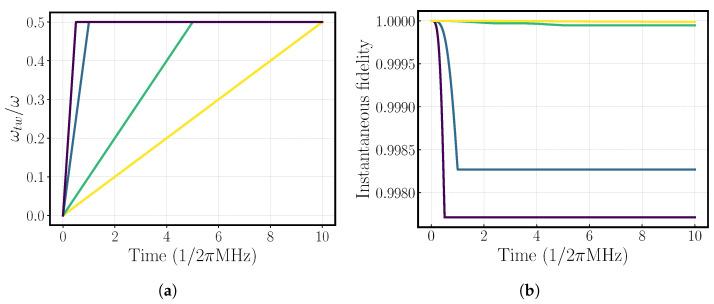
Linearly ramping the tweezer power to $\omega_{\mathrm{tw}}=0.5\omega$ for various ramp times $\tau_{\mathrm{ramp}}=\left(0.5,1,5,10\right)/\omega$. The ramp profiles are plotted in (**a**). In (**b**), the instantaneous fidelity with the ground state is plotted. The fidelity decays as the ramp speed is increased. However, all four ramps produce the new ground state with high fidelity (>99%). Assuming a trapping frequency ω=2π MHz, the slowest ramp considered here corresponds to a time of ∼1.6 μs, one order of magnitude higher than the trap period. All the others stay under this value and guarantee fidelity with up to $10^{-4}$ accuracy.

**Figure A3:**
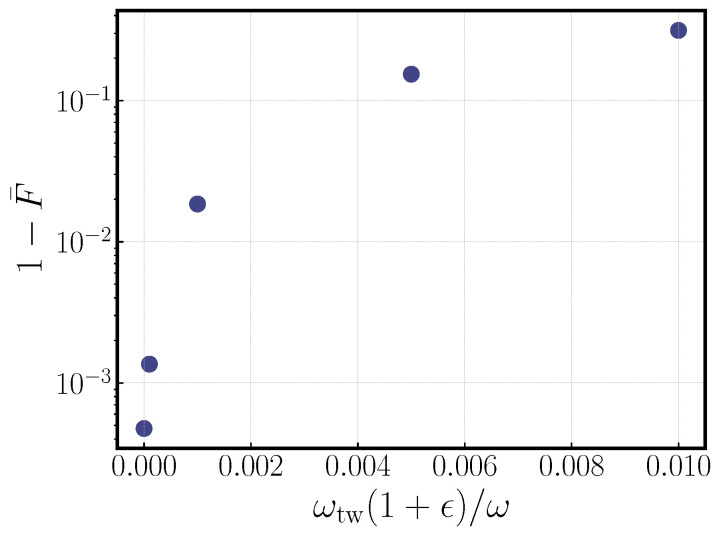
The decay in the mean average gate fidelity upon the introduction of frequency drifts, ϵ, around the expected tweezer frequencies, $\omega_{\mathrm{tw}}$.

**Figure A4:**
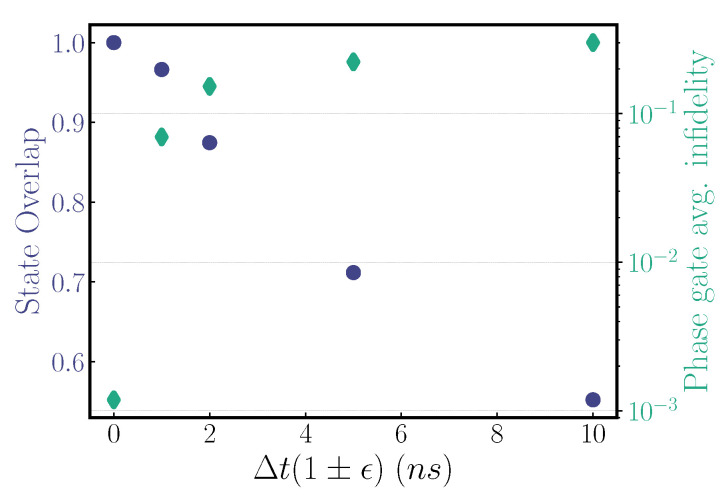
An analysis of timing error sensitivity for the developed scheme. The purple axis quantifies the decoupling between the motional and internal states of the ions, showing the overlap between the initial and final motional states. And the green axis quantifies how well the obtained unitary still corresponds to the target unitary.

**Figure A5:**
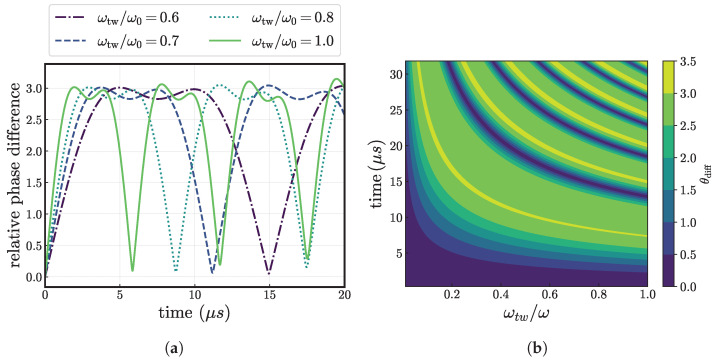
An analysis of the zero-point energy dephasing contribution (quantified as the relative phase difference, $\theta_{\mathrm{diff}}$) as a function of time (**a**) and tweezer frequency (**b**) in the range of tens of microseconds. Higher tweezer frequencies are observed to have a faster rephasing period.

## Data Availability

The datasets are available on request from the authors.

## Abbreviations

The following abbreviations are used in this manuscript:

ZPE	Zero-point energy
COM	Center-of-mass

## Appendix A. New-Mode Coherent State Preparation

In the main text, we assume the tweezers are retained during state preparation and throughout the whole gate, meaning that we work on a tweezer-modified coherent state basis. In this section, we investigate a scenario where state preparation to the ground state is performed with the tweezers off, and only then is the tweezers’ intensity ramped up adiabatically. We observe that this enables high-fidelity preparation of the new-mode coherent states without significantly compromising on the gate time.

In Figure A1, we plot the spectrum of $H_{0}+H_{\mathrm{tw}}$, which describes the motion of the ions including the optical tweezers. At $\omega_{\mathrm{tw}}/\omega =0$, the three lowest eigen-energies are $E_{0}=\frac{1}{2}\left(1+\sqrt{3}\right)\omega$, $E_{1}=\frac{3}{2}+\frac{1}{2}\sqrt{3}\omega$, and $E_{2}=\frac{1}{2}+\frac{3}{2}\sqrt{3}\omega$, as expected. Turning on the tweezers increases the energies of all states, with the energy gaps changing due to squeezing, which occurs given the changing frequency of the harmonic oscillator. We see that the largest gap is between the ground state and the first excited state, suggesting that the best state to adiabatically ramp from is the ground state.

In Figure A2a, we linearly ramp the tweezer trapping frequency $\omega_{\mathrm{tw}}$, with the corresponding instantaneous fidelity with the ground state shown in Figure A2b. Switching on the tweezers after ground state preparation yields a ground state overlap higher than 0.998 with the tweezer ramping times under a trap period. For a higher fidelity, ramping as slow as for five trapping periods yields a fidelity above 0.9995, with this time representing less than one microsecond added to the overall gate time.

## Appendix B. The Gate Performance with Tweezer Stability

In the main text, we assume a fixed and controlled tweezer frequency. However, in a real-life implementation, tweezer drifts can affect the gate performance and cause a decay in the gate fidelity.

We study how much this affects the proposed gate by determining the average mean infidelity of all of the two-ion solutions in Figure 2 with a fidelity above 0.95 and determining how this average mean would behave when small drifts, ϵ, were added to each gate’s expected tweezer frequency; see Figure A3.

We observe that the drifts should be under $\epsilon ∼0.001/\omega_{z}$ in order to minimize the compromise in the gate performance. This means that if we were to have an ordinary axial trap frequency of 500 kHz, then our tweezer trap frequency could not vary by more than 500 Hz.

We consider the origin of such instability in the tweezer frequency. More specifically, the tweezer trap frequency scales as $\omega_{\mathrm{tw}}=-4U_{\mathrm{dip}}/(m\omega_{0}^{2}$) [16], and the dipole moment Udip∝Re{α}I, with the Gaussian beam intensity $I\approx 2P/(\pi \omega_{0}^{2})$ [27]. We then obtain the following relation:

(A1) $$\omega_{\mathrm{tw}}=-\frac{8P}{\pi m\omega_{0}^{4}}.$$

As the polarizability is only dependent on the atom species and light polarization, the biggest source of instability in the tweezer frequency will be the beam power (linearly dependent) and the beam waist (quartically dependent).

## Appendix C. Sensitivity to Timing Errors

We wish to gauge how sensitive this scheme is to imprecision in the timing of the sequence kicks. We add/subtract small delays (in the order of a few nanoseconds) according to a normal distribution to/from all of the kicks in the two-ion solutions presented in Figure 2 with a fidelity above 0.95 and observe how this affects the gate performance. As this timing change affects the decoupling of the motional and internal states, besides looking at how well we performed the desired phase gate (the average phase gate infidelity), we also look at how the final acquired state overlaps with the initial state, as a measure of how well the motion and spin have decoupled; see Figure A4. We conclude that the scheme is too sensitive for timing errors above 2 ns.

## Appendix D. Zero-Point Energy Rephasing Points

In order to study the relative phase difference between the three different spin-states, we consider the spin-dependent zpe phase:

(A2) $$\begin{array}{c} \theta^{\cdot \cdot }=\exp i(\omega_{c}^{\cdot \cdot }+\omega_{s}^{\cdot \cdot })t. \end{array}$$

The relative phase differences are studied by plotting $\theta_{\mathrm{diff}}=|\theta^{↑↑}-\theta^{↑↓}\left|+\right|\theta^{↑↑}-\theta^{↓↓}|$ for different tweezer frequencies; see Figure A5.

When $\theta_{\mathrm{diff}}=0$, the three spin-dependent phases are the same and have thus re-phased. Higher tweezer frequencies show a shorter rephasing period; however, we do not observe complete rephasing like in t=0. This means that in principle, we could start the gate at any motional state and wait for an approximate rephasing, but the small phase difference remaining will limit the attainable fidelities.

## Appendix E. Spin-Dependent Phase Factors

The acquired phase from the kick unitary has been defined as $\xi =\sum_{m}\left(-\theta_{N}^{\cdot \cdot }/2+\Theta_{m}^{\cdot \cdot }+\Xi_{m}^{\cdot \cdot }\right)$, with

(A3) $$\begin{array}{cc} \Theta_{m}^{\cdot \cdot } & =-\sum_{n=2}^{N}\sum_{k=1}^{n-1}p_{m,n}^{\cdot \cdot }p_{m,k}^{\cdot \cdot }\sin\left(\omega_{m}^{\cdot \cdot }\Delta t_{kn}\right)\;,\;p_{mk}=z_{k}l_{m}^{\cdot \cdot }b_{m}^{\cdot \cdot }, \end{array}$$

(A4) $$\begin{array}{cc} \; & =-\sum_{n=2}^{N}\sum_{k=1}^{n-1}z_{n}z_{k}\left[{l_{m}^{\cdot \cdot }}^{2}{\left(b_{m}^{\cdot \cdot }\cdot 1_{2}\right)}^{2}\sin\left(\omega_{m}^{\cdot \cdot }\Delta t_{kn}\right)\right]\;,\;l_{m}^{\cdot \cdot }=\sqrt{\frac{\hbar }{2M\omega_{m}^{\cdot \cdot }}}. \end{array}$$

And with

(A5) Ξm··=−∑n=2NReαm∑k=1Npm,k··e−iθn··,θn··=ωm··∑j=1nτj=ωm··tn.

For two ions, the mode vectors are obtainable analytically. This enables us to write the phase factors as

(A6a) $$\begin{array}{cc} \Theta^{\cdot \cdot } & =\Phi_{-}^{\cdot \cdot }+\Phi_{+}^{\cdot \cdot }1,\;\Xi^{\cdot \cdot }=\Gamma^{\cdot \cdot },\;\cdot \cdot =\left\{↑↑,↓↓\right\}, \end{array}$$

(A6b) $$\begin{array}{cc} \Theta^{\cdot \cdot } & =\Lambda^{\cdot \cdot }+\Phi_{+}^{\cdot \cdot }1,\;\Xi^{\cdot \cdot }=\Upsilon^{\cdot \cdot },\;\cdot \cdot =\left\{↑↓,↓↑\right\}, \end{array}$$

where we have dropped the *m* subscript on Θ for convenience and defined $\beta_{1}=\sqrt{1+{\bar{w}}^{4}}-{\bar{w}}^{2}$, $\beta_{2}=-\sqrt{1+{\bar{w}}^{4}}-{\bar{w}}^{2}$, $\bar{\omega }=\omega_{\mathrm{tw}}/\omega$. We also introduce the terms

(A7a) $$\begin{array}{cc} \Phi_{\pm }^{\cdot \cdot } & =-\frac{\hbar }{2M}\sum_{n=2}^{N}\sum_{k=1}^{n-1}z_{n}z_{k}\left[\frac{1}{{\tilde{\omega }}_{1}^{\cdot \cdot }}\sin\left({\tilde{\omega }}_{1}^{\cdot \cdot }\Delta t_{kn}\right)\pm \frac{1}{{\tilde{\omega }}_{2}^{\cdot \cdot }}\sin\left({\tilde{\omega }}_{2}^{\cdot \cdot }\Delta t_{kn}\right)\right], \end{array}$$

(A7b) $$\begin{array}{cc} \Lambda^{↑↓} & =-\frac{\hbar }{M}\sum_{n=2}^{N}\sum_{k=1}^{n-1}z_{n}z_{k}\left[\frac{1}{{\tilde{\omega }}_{1}^{\cdot \cdot }}\chi_{1}\sin\left({\tilde{\omega }}_{1}^{\cdot \cdot }\Delta t_{kn}\right)+\frac{1}{{\tilde{\omega }}_{2}^{\cdot \cdot }}\chi_{2}\sin\left({\tilde{\omega }}_{2}^{\cdot \cdot }\Delta t_{kn}\right)\right], \end{array}$$

(A7c) $$\begin{array}{cc} \Lambda^{↓↑} & =\frac{\hbar }{M}\sum_{n=2}^{N}\sum_{k=1}^{n-1}z_{n}z_{k}\left[\frac{1}{{\tilde{\omega }}_{1}^{\cdot \cdot }}\chi_{2}\sin\left({\tilde{\omega }}_{1}^{\cdot \cdot }\Delta t_{kn}\right)+\frac{1}{{\tilde{\omega }}_{2}^{\cdot \cdot }}\chi_{1}\sin\left({\tilde{\omega }}_{2}^{\cdot \cdot }\Delta t_{kn}\right)\right], \end{array}$$

(A7d) Γ··=−ℏM∑n=2N∑k=1NRezkα1ω˜1··(σ1z+σ2z)eω˜1··tk,

(A7e) Υ↑↓=−ℏ2M∑n=2N∑k=1n−1Rezkα1ω˜1↑↓(β1−1)ζ1eω˜1↑↓tk+zkα2ω˜2↑↓(β2−1)ζ2eω˜2↑↓tk,

(A7f) Υ↓↑=−ℏ2M∑n=2N∑k=1n−1Rezkα1ω˜1↓↑(β2+1)ζ2eω˜1↓↑tk+zkα2ω˜2↓↑(β1+1)ζ1eω˜2↓↑tk,

where $\chi_{i}=\beta_{i}/\left(1+\beta_{i}^{2}\right)$ and $\zeta_{i}=\sqrt{1+\beta_{i}^{2}}$.

## Appendix F. State-Dependent Motional Eigenmodes upon the Introduction of Optical Tweezers into the System

We determine the motional eigenmodes by following the process described in [28]. They are determined analytically by diagonalizing the system’s Hamiltonian:

(A8) $$\begin{array}{c} H=\frac{M}{2}\sum_{n=1}^{N}{\left(\dot{q_{n}}\right)}^{2}-\frac{1}{2}\sum_{m,n=1}^{N}q_{m}q_{n}{\left.\left(\frac{\partial^{2}V}{\partial x_{m}\partial x_{n}}\right)\right|}_{q_{m,n}=0}\;, \end{array}$$

with terms representing the trap and the Coulomb repulsion between ions. Here, the Coulomb potential $V(q_{1},...,q_{N})$ was replaced with its second derivative with a minimal loss of accuracy. This is possible considering that the variables $q_{m}=x_{m}-x_{m}^{\left(0\right)}$ are much smaller than the distance between equilibrium positions $x_{m}^{\left(0\right)}$, which means that the potential resembles its second derivative around these points.

We then determine the Hessian matrix of our system, $A_{nm}$, with which we can extract the system’s eigenvalues; see Table A1.

**Table A1:** The phonon mode frequencies and eigenvectors for the different spin-state combinations upon the introduction of tweezers into the system, $\bar{\omega }=\omega_{\mathrm{tw}}/\omega$. $\beta_{i}$ is defined as $\beta_{1}=\sqrt{4+{\bar{\omega }}^{4}}-{\bar{\omega }}^{2}$, $\beta_{2}=-\sqrt{4+{\bar{\omega }}^{4}}-{\bar{\omega }}^{2}$.

	${\left({\tilde{\omega }}_{1}^{\cdot \cdot }/\omega \right)}^{2}$	${\left({\tilde{\omega }}_{2}^{\cdot \cdot }/\omega \right)}^{2}$	${\tilde{b}}_{1}$	${\tilde{b}}_{2}$
|↓↓〉	1	3	$\frac{1}{\sqrt{2}}\left(\begin{array}{c} 1\\ 1 \end{array}\right)$	$\frac{1}{\sqrt{2}}\left(\begin{array}{c} -1\\ 1 \end{array}\right)$
|↑↑〉	$1+{\bar{\omega }}^{2}$	$3+{\bar{\omega }}^{2}$	$\frac{1}{\sqrt{2}}\left(\begin{array}{c} 1\\ 1 \end{array}\right)$	$\frac{1}{\sqrt{2}}\left(\begin{array}{c} -1\\ 1 \end{array}\right)$
|↑↓〉	$\frac{1}{2}\left(4-\beta_{1}\right)$	$\frac{1}{2}\left(4-\beta_{2}\right)$	$\frac{1}{\sqrt{4+|\beta_{1}{|}^{2}}}\left(\begin{array}{c} \beta_{1}\\ 2 \end{array}\right)$	$\frac{1}{\sqrt{4+|\beta_{2}{|}^{2}}}\left(\begin{array}{c} \beta_{2}\\ 2 \end{array}\right)$
|↓↑〉	$\frac{1}{2}\left(4-\beta_{1}\right)$	$\frac{1}{2}\left(4-\beta_{2}\right)$	$\frac{1}{\sqrt{4+|\beta_{2}{|}^{2}}}\left(\begin{array}{c} -\beta_{2}\\ 2 \end{array}\right)$	$\frac{1}{\sqrt{4+|\beta_{1}{|}^{2}}}\left(\begin{array}{c} -\beta_{1}\\ 2 \end{array}\right)$
